# Barriers and enablers to the implementation of a complex quality improvement intervention for acute kidney injury: A qualitative evaluation of stakeholder perceptions of the Tackling AKI study

**DOI:** 10.1371/journal.pone.0222444

**Published:** 2019-09-20

**Authors:** Laura Lamming, Eileen McDonach, Mohammed A. Mohammed, John Stoves, Andy J. Lewington, Russell Roberts, Yohan Samarasinghe, Nikunj Shah, Richard J. Fluck, Natalie Jackson, Melanie Johnson, Carol Jones, Nicholas M. Selby

**Affiliations:** 1 Faculty of Health Studies, University of Bradford, Bradford, West Yorkshire, United Kingdom; 2 Bradford Teaching Hospitals NHS Foundation Trust, Bradford, West Yorkshire, United Kingdom; 3 Leeds Teaching Hospitals NHS Trust, Leeds, West Yorkshire, United Kingdom; 4 The NIHR Leeds In Vitro Diagnostics Co-operative, Leeds, West Yorkshire, United Kingdom; 5 Bradford Institute for Health Research, Bradford, West Yorkshire, United Kingdom; 6 Frimley Health NHS Foundation Trust, Frimley, United Kingdom; 7 Ashford and St Peter’s NHS Foundation Trust, Surrey, United Kingdom; 8 Department of Renal Medicine, Derby Teaching Hospitals NHS Foundation Trust, Derby, United Kingdom; 9 Yorkshire & Humber AHSN improvement academy, Bradford Institute for Health Research, Bradford, West Yorkshire, United Kingdom; 10 Centre for Kidney Research and Innovation, School of Medicine, University of Nottingham, Derby Teaching Hospitals NHS Foundation Trust, Derby, United Kingdom; University of Central Lancashire, UNITED KINGDOM

## Abstract

**Background:**

Acute kidney injury in hospital patients is common and associated with reduced survival and higher healthcare costs. The Tackling Acute Kidney Injury (TAKI) quality improvement project aimed to reduce mortality rates in patients with acute kidney injury by implementing a multicomponent intervention comprising of an electronic alert, care bundle and education in five UK hospitals across a variety of wards. A parallel developmental evaluation using a case study approach was conducted to provide the implementation teams with insights into factors that might impact intervention implementation and fidelity. The qualitative element of the evaluation will be reported.

**Methods:**

29 semi-structured interviews with implementation teams across the five hospitals were carried out to identify perceived barriers and enablers to implementation. Interviews were taped and transcribed verbatim and Framework analysis was conducted.

**Results:**

Interviews generated four ‘barriers and enablers’ to implementation themes: i) practical/contextual factors, ii) skills and make-up of the TAKI implementation team, iii) design, development and implementation approach, iv) staff knowledge, attitudes, behaviours and support. Enablers included availability of specialist teams (e.g. educational teams), multi-disciplinary implementation teams with strong leadership, team-based package completion and proactive staff. Barriers were frequently the converse of facilitators.

**Conclusions:**

Despite diversity of sites, a range of common local factors–contextual, intervention-based and individual–were identified as potential barriers and enablers to fidelity, including intervention structure/design and process of/approach to implementation. Future efforts should focus on early identification and management of barriers and tailored optimisation of known enablers such as leadership and multidisciplinary teams to encourage buy-in. Improved measures of real-time intervention and implementation fidelity would further assist local teams to target their support during such quality improvement initiatives.

## Introduction

Acute Kidney Injury (AKI) in hospitalised patients is common and associated with higher mortality rates as well as healthcare costs [1]. The Tackling AKI (TAKI) study was a quality improvement project in five UK hospitals selected for diversity of characteristics, over an eighteen-month period. The intervention was designed to be hospital-wide, and both long and short-stay wards across a range of specialities were involved including: respiratory, medical admissions, acute medical unit, elderly admissions/medicine care, vascular, orthopaedics, and high dependency. The aim was to reduce mortality rates in patients with AKI by implementing a locally tailored, multi-component package of interventions to improve basic AKI care within a pragmatic stepped-wedge trial design [2]. Interventions consisted of three main components (i) an electronic AKI detection and alerting system (e-alert), (ii) a care bundle (typically a small set of evidence-based practices that when performed improve patient outcomes [3]) and (iii) education. AKI detection was based on biochemistry results and enacted through a nationally mandated algorithm. The subsequent alert was displayed on ward computers or multi-media screens to flag patients with AKI to clinical staff and describe AKI severity (AKI stage 1, 2 or 3). The care bundle outlined the basic steps for assessment, investigation and management of a patient with an AKI and maintained a record of the care completed, reminding staff of optimal care practices and ensuring care completion. The education package for clinical staff aimed to raise awareness and knowledge of AKI and its treatment, and could be delivered in a variety of mediums such as verbally, with booklets or by other materials such as posters or screensavers. Although examples were provided, interventions and their implementation were developed and tailored to each hospital’s context by multidisciplinary (MDT) implementation teams. This meant that each of the three intervention components were present but different in some elements of presentation and content. Local teams were supported by well-established quality improvement practices including initial learning events with representatives from each hospital and peer assist and review meetings during implementation with staff from all hospitals. This incorporated a robust challenge of plans and confirmation of action process. These events maximised opportunities for shared learning between the five hospitals. Development of communication plans, senior staff engagement, and methods to sustain change by reinforcing behaviours through the review of the project, success celebrations and group/individual recognition were also implemented.

The TAKI project was evaluated using both summative and developmental approaches. The summative (quantitative) evaluation and outcomes have been reported elsewhere [4]. Briefly, the quantitative evaluation found that the TAKI intervention did not alter 30-day AKI mortality, but did result in reductions in AKI duration and length of stay, accompanied by improvements in quality of care, varying between hospitals. In addition, AKI incidence increased, likely reflecting improved recognition. The developmental evaluation used a case-study approach (within and between hospitals) and aimed to provide the implementation teams with insights into (both local and common) factors that might impact intervention fidelity, defined as the extent to which the intervention was delivered as intended, so that they could address these issues as required. A developmental evaluation method was therefore used with regular feedback. A major aspect of the qualitative evaluation was to explore stakeholder perceptions of barriers and enablers to implementation of the TAKI project across the five hospitals through 1:1 interviews with key stakeholders involved.

## Methods

The TAKI project was considered service evaluation by Derbyshire Research Ethics Committee and the developmental evaluation was subsequently approved by each hospital. Ethical approval for the qualitative interviews was provided by the chair of the Biomedical, Natural, Physical and Health Studies Research Ethics Panel at the University of Bradford.

The Consolidated Criteria for Reporting Qualitive Studies (COREQ) checklist was used to ensure comprehensive reporting [5].

### Interviews

Stakeholders included members of the TAKI implementation team at each of the five hospitals.

After providing informed, written consent, team members took part in a semi-structured, one-to-one interview in person at the hospital or by telephone. A purposive sampling strategy was used to invite team members by personal email (from LL) from the following roles: project manager, lead clinician and/or nurse, lab technician or IT specialist, junior doctor or nurse, pharmacist, education or quality improvement specialist, patient and public representative. Where member specialities overlapped, the first respondent was chosen. Not all specialities were available at each hospital. Two hospitals in the same city involved the same project team members, intervention design and implementation. For the purposes of this evaluation, they were considered as a single hospital for these interviews. The Chief Investigator (CI) was interviewed to provide an overview of the project across the hospitals. Due to the developmental approach of the evaluation, LL had already met a large proportion of the participants through learning events and other data-collection processes, and they were aware of the purpose of the evaluation.

Interviews were conducted three to six months post-implementation by a trained, female post-graduate Research Fellow with experience in qualitative data collection for health research (LL). Each interviewed lasted approximately 45–60 minutes. An interview schedule (S1 File) was used and consisted of eight to thirteen questions depending on the role of the team member (e.g. clinical leads answered more questions based on their early high-level involvement with the project). One question asked specifically about barriers and enablers to implementation although it was expected that other questions would contribute additional data. Field notes were also made during interviews.

Interviews were recorded, transcribed and Framework Analysis was conducted to identify themes and patterns in the data using QSR NVivo 10 [6–8]. Documentation (five transcripts and/or minutes, notes and slides from cross-hospital project meetings) was collected to supplement the interviews and was included in analysis. A thematic framework was developed, including an overarching ‘barriers and enablers’ theme derived deductively from the evaluation question, with subthemes formed using an inductive approach. An independent second researcher reviewed a proportion of the data during initial indexing (two interviews, one meeting transcript) and interpretation (10% of charted data) to identify additional or different themes. This resulted in only minimal changes to subthemes, including how they were described. Although analysis by staff specialty and hospital was possible, themes are reported in aggregate to avoid violating anonymity with small sample sizes. It also reassured participants that they could discuss negative perceptions or events openly. This protects localised anonymity or ‘internal confidentiality’—ensuring those who have been involved in the evaluation (‘insiders’) can’t recognise fellow TAKI team members, as well as ‘external confidentiality’- preventing the possibility that TAKI team members may be identified by other staff or patients from their employing hospital [9]. Transcripts were not returned to participants for comment or correction before analysis and member checking did not occur post-analysis.

## Results

Twenty-nine interviews (range per hospital 5 to 9), including one with the CI, were conducted. Six additional team members were invited, but did not participate either because they failed to reply, felt their involvement was too limited, or their specialty overlapped with another team member who had already provided an interview. Participants included Project Managers (n = 4), Lead clinicians or nurses (n = 7), Lab technicians (n = 3), other frontline doctors or nurses (n = 6), pharmacists (n = 3), education or QI specialists (n = 4), patient, public involvement representatives (n = 1) and the CI (n = 1). Due to challenges in reaching some team members and varying sizes and make-up of implementation teams, representatives from each of the aforementioned roles were not always present for each hospital. Therefore the number of interviews was restricted based on availability and inclusion of certain roles. Saturation was achieved for the majority of questions both within and across sites, with the main difference being additional role-specific details when describing package components (e.g. technical specifications) or additional insights from team members who were only present in a single site.

Four themes relating to ‘barriers and enablers’ to implementation were identified across all hospitals, i) practical and contextual factors, ii) the TAKI implementation team, iii) the design, development and implementation approach, iv) staff knowledge, attitudes, behaviours and support (see S2 File for subthemes).

### Practical and contextual factors

Interviews from all hospitals indicated that implementation was facilitated by a range of existing resources including staff teams and IT systems. Availability, or knowledge, of certain specialist teams was perceived to be beneficial, for example, education teams were well utilised, where available, and reported to reduced implementation team burden.

*“…the things that have really made a difference between the different centres was whether or not there were established teams to actually go out and target ward staff either from an educator’s point of view or from a sort of quality and patient safety point of view*. *The hospitals that had those teams already established and in place were very much more set up and able to do that than those that weren’t.”* PN:28

The ICE pathology reporting system was used by every hospital and was perceived as easy to configure without outside support. Additional resources, such as the presence of project funding were also acknowledged as greatly influential in facilitating implementation across all hospitals as it allowed for innovative intervention design and prompt implementation as well as adding credibility.

Staff familiarity for different components varied between hospitals, with two hospitals having little experience of care-bundle forms and one with no experience of e-alerts (NB the detection algorithm could be installed in the biochemistry laboratory software, but results were not released to healthcare providers until the implementation phase at that hospital). Where hospitals were already familiar and/or had had success with e-alerts, care bundles and education implementation previously, this was an enabler, as staff already understood the format and could engage with the new tools quickly. Senior staff support and hospital-wide attitudes such as perceived importance of quality improvement was also reported to enable implementation by preventing or quickly problem-solving system-based barriers.

Reported barriers to implementation were typically the converse of the enablers (S2 File, subthemes 1–13)

### The TAKI implementation team

Every hospital had a multi-disciplinary implementation team, and staff across all sites perceived this as an enabler (Project Managers, Clinical Leads, Pharmacists, Lab technicians, nurses, Education specialists and Quality Improvement specialists) as the mix of skill-sets reduced the chances of staff performing or contributing to tasks they were inexperienced with, such as project management. In addition, many of the teams had members with existing links to relevant networks or had been involved in prior AKI work before joining the team, prompting informed and quick development of intervention content.

A variety of team members across all hospitals including the CI, identified a variety of team characteristics that were perceived as enablers. Where the members bought in to the intervention (most frequently cited by Project Managers n = 3), were proactive and cohesive (most frequently cited by Clinical Leads, n = 5), met regularly and consistently (many team members fluctuated in their involvement which was more acceptable in larger teams, but problematic in the very small team that existed at one hospital), and had strong leadership and influence (most frequently cited by Project Managers, n = 3), facilitated implementation. In particular, while some hospitals were able to use the dedicated resource time allotted for the project, others struggled to do so and this was perceived as a significant facilitator (or barrier when absent).

*“…the enthusiasm of the clinical leads we’ve got is a rare event… so a clinician who can influence other clinicians is like hens’ teeth*, *they usually can influence with their own field*, *but influencing outside their own field is really difficult so it takes someone with a very specific type of communication skills to be able to do that and fortunately we’ve got people in the team that can do that*, *who are good influencers*, *and that’s been hugely necessary and valued really within the work…”* PN:1

Barriers were often the counter of the enablers, but also included the perception that the team had no authority (mentioned by at least one participant at each hospital including Project Managers and Clinical Leads), as well as members who were unconvinced by the TAKI project itself (also mentioned by at least one staff at each site, including nurses, Project managers, PPI representatives, or doctors) (S2 File, subthemes 14–29).

### Design, development and implementation approach

All hospitals cited approaches that facilitated staff-ownership, engagement or a team-based approach to the package and AKI management, or linked to other relevant initiatives, as enabling implementation, with one hospital’s staff (either Project Manager, Clinical Lead, pharmacist or Lab technician) repeatedly citing all four. Three hospitals cited a tailored approach, including preparatory work with teams before and during spread as well as feedback on compliance (for example the ratio of completed care bundles to identified AKIs) as helping, with the latter mentioned most frequently and by a variety of staff. Some hospitals had more time for this than others, depending on where they came in the stepped-wedge order of implementation, but attempts to engage relevant staff occurred at all hospitals with varying levels of success. One hospital suggested staff may have been engaged more successfully with more face-to-face recruitment.

Specific characteristics of local intervention packages were also perceived to be important facilitators of implementation, for example staged e-alerts (indicating stage of AKI as well as its presence) made identification of AKI severity easier, stickered and brief care bundles made completion easier (core actionable care items listed on the care-bundles varied from eight to thirty-nine, across the five hospitals), and education focusing on AKI complexities rather than just how to use package components was identified as being helpful by team leads in all but one hospital.

Persistent, multiple and tailored attempts to promote the project with different groups of frontline staff, including the use of a variety of additional materials (stationery, presentations, websites etc.) were perceived as enablers. However, the variety and volume of additional materials varied between hospitals with some giving away popular ‘freebies’ (branded stationery, water bottles etc.) as well as providing alternative education or recording materials. While freebies were well received, especially credit-card sized quick-reference cards for nurses, substantial documents like ward books for recording alerts were rarely used and difficulties were often encountered when trying to add electronic materials to local websites (e.g. journal articles, British National Formulary manual).

Quality improvement methods (cited by Project Managers at two hospitals), promoting the utility of the package (cited by a Project Manager and an Education Specialist at two hospitals, although staff at all hospitals reported that they perceived usefulness of the package themselves or for other frontline staff), senior executive support (highlighted by a range of staff from two hospitals in particular) and inter-hospital learning (either via official peer review or assist events, or informal contact) were also reported to contribute to successful implementation.

In contrast, hospitals struggled to reach some groups of frontline staff (doctors were often cited, due to their regular ward rotation), but mostly barriers were identified with the intervention package itself, for example, inappropriate or insufficient content or suggesting care could only be carried out by one staff group despite perceived ability of other groups to be able to perform the same care (S2 File, subthemes 30–54). Only one hospital explicitly included staff members beyond doctors in enactment of the items on the care-bundle checklist.

*“The one thing I find a little frustrating is that it’s [care bundle] very medically focussed and I think that we have a lot of advanced nurse practitioners… that I think could sign-off some of this stuff.”* PN:14

### Staff knowledge, attitudes, behaviours and support

Proactive frontline, senior or executive staff who championed the project on behalf of the implementation team were seen by numerous participants across all hospitals as enablers. While nurses who were interviewed typically identified nursing staff as enablers due to their static presence on wards (unlike doctors who have 3–4 monthly rotational posts), other staff across all hospitals highlighted how involved and important nurses were in the implementation, describing various activities they performed and their general enthusiasm and commitment to the project.

*“I think it’s really good that we’re getting into the nursing profession*, *they’re really embracing it because they will be the framework I think around … the doctors will change again won’t they in August*, *so I think the more we can embed it there*, *will start to seep through into others*. *We would like to make a bigger impact right now wouldn’t we*, *but I think we’re feeling these sorts of things take a while to change a whole culture*, *but if the nurses are taking it on as a cultural change I’m really encouraged by that longer term.”*PN:34b

The inclusion of the MDT in the package facilitated staff from many different roles (e.g. nurses, pharmacists) to prompt colleagues from other disciplines to enact the package in real-time.

Conversely, some staff failed to see the benefit of the project, didn’t see use of the package as part of their role and/or senior staff could be problematic if they negatively influenced junior staff behaviour.

*“I think the problem comes where*…*because obviously a lot of us*, *we are newer doctors*, *and we are much more open to change*, *and we don’t have expectations that have been there for years*, *whereas in a lot of the older doctors*, *not necessarily consultants*, *but registrars and more senior SHOs who are people from their second year up until their fifth year*, *in those people they have been practicing medicine for quite a lot longer*, *and maybe don’t feel as though the bundle is necessary.”* PN:7

In addition, some staff used physical ward structures (e.g. not routinely attending to ward whiteboards that show updating patient information) in unexpected ways that inhibited take up of the package (S2 File, subthemes 55–65).

## Discussion

### Main findings

Implementation teams reported four types of barriers and enablers that could have influenced fidelity of the AKI package of interventions: practical and contextual characteristics of the hospital, the skills and composition of the TAKI implementation team, the design and implementation of the package and the attitudes, behaviours and knowledge of hospital staff. These findings suggest that hospital and project resources and experiences were important, but also constraining in terms of the flexibility, creativity and reach of the intervention implementation. Relatively large, multidisciplinary teams with relevant knowledge, time and skills were key to implementation, but could be derailed by poor leadership and lack of belief in the project. Similarly, frontline staff did not always see the benefit of the project. Buy-in could be facilitated however, by the approach to development and implementation of the intervention components themselves, especially by involving frontline staff in iterative design and demonstrating staff-informed changes. Unfortunately the importance of the multidisciplinary team was not always reflected in the design of the intervention components, which inhibited implementation. However short, simple components and a persistent and responsive approach to implementation helped. Across all themes, staff were highlighted as key to the implementation of the intervention, either in terms of the implementing team, frontline staff, or senior executives. Characteristics, behaviours, availability, buy-in and attitudes to intervention components of those directly or indirectly involved with the intervention package comprised more than half of the barrier and enabler subthemes.

### Context of literature

The barriers and enablers to implementation identified in the TAKI project are not unfamiliar to quality improvement researchers with context, cultures, capacity, intervention usability, staff engagement/resistance and leadership featuring prominently in the literature [10–14]. In fact, The Health Foundation identify many of these as areas where quality improvement approaches should focus [15]. These factors have been conceptualised into a model for ‘understanding success’ of implementation [16], and suggestions have been made for how to address them [10].

Although these findings are common, they reinforce the need to identify early and address local factors (especially staff) before and during implementation, and highlight the difficulty in overcoming them. Despite the use of well-established quality improvement approaches and extensive consideration of implementation prior to the start of the TAKI study, these problems still arose. The literature demonstrates that attempts are being made to translate such recommendations into practice [17], but evidence for the effectiveness of these attempts is mixed [18]. This may be due to a lack of knowledge of the optimal way to identify *important* barriers [18]. The TAKI study offers some support for this as the implementation teams did identify characteristics of the hospitals at the start of the project to inform implementation (e.g. number of beds, number of staff, and presence of onsite nephrology support). They also used many of the influencing techniques mentioned in Gollop et al., (2004) [14] to address staff engagement (e.g. presenting data, demonstrating tools, persistence). However, given the results, it suggests that such approaches may be insufficient to capture or address the range of influential factors.

### Strengths and limitations

There were some limitations to the evaluation. Interviewees were asked to provide accounts about themselves and their colleagues, which may have been subject to social desirability bias [19] when discussing themselves, or the halo effect [20] when discussing their team. However, attempts were made to address these potential biases by interviewing a range of team members in order to triangulate data and identify inconsistencies. In addition, between-hospital data were not explicitly reported for this analysis to ensure participants had anonymity and confidentiality in internal and external reports.

### Implications

There are some implications that could be inferred from our results, when combined with findings from the quantitative evaluation. While the quantitative evaluation showed a lack of significant change in mortality rates, care bundle completion and process measures such as care-practices promoted by the bundles showed an improvement with the intervention, but also demonstrated variation in the degree of improvement between hospitals. Although no obvious patterns between our results and process outcome variations are present on observation, these variations are likely to be a product of the interaction between barriers and enablers in each site. Further improvements in processes of care could eventually result in larger improvements in patient outcomes across all hospitals. Further work exploring patterns of fidelity between hospitals could be beneficial. Hospitals were selected for their diverse characteristics, and the three intervention components were tailored to the local requirements of each hospital to promote uptake. Systematic mapping of these contexts and specific adaptations may also give additional useful insights about intervention fit and appropriateness of local adaptations and implementation strategy [21].

The qualitative findings present four additional implications:

#### Identifying barriers

Preparatory work with prospective implementation teams prior to implementation could help to identify potential issues, develop the intervention and tailor the support. This could be based on the Theoretical Domains Framework (TDF) [22] through survey, interviews or group discussions. The TDF is a framework of evidence-based constructs that explain behaviour change, with the aim of supporting implementation of interventions. Literature suggests that evidence-based approaches to behaviour change in quality improvement are preferable and optimise implementation or at least facilitate easier identification of reasons for failure [23,24]. Re-measuring and refining the intervention, in response to emergent findings, at regular time points is also advised. Secondly, fidelity measures are a key component of implementation and can identify areas that need attention. This may offer important insights in refining the TAKI theory of change, defined as how the package acts on individuals and systems to exact the desired change, identifying key ‘active ingredients’ or enablers which contribute to improved outcomes, and where scarce resources may be best targeted in the future.

#### Targeting barriers

The COM-B model [25,26] could be a potentially accessible model for implementation teams to use. This would map findings onto three domains thought to be required to enable behaviour change–capability, opportunity and motivation. The advantages of this model are that it incorporates the TDF, is fairly straightforward to use and could be used at an early stage to identify and prioritise barriers to address. Currently, reported barriers in this study could be interpreted as staff struggling with all three domains, however application of the TDF could illuminate more specific sub-domains that need attention and allow for more efficient targeting of barriers.

#### Optimising enablers

Careful consideration of implementation team members is advisable, beyond those that are just willing. Strong, enthusiastic leaders (with perceived influence) should be recruited early on to support the selection of the implementation team and maintain momentum. Leaders should be able to advocate for and continue implementation during longer-term changes such as the yearly intake of new doctors. Multi-disciplinary teams are important for ensuring tailoring, acceptability and efficiencies in intervention components and task completion. Specifically, a range of staff should be enabled to use the multicomponent intervention in future. This should facilitate promotion of the intervention across a range of disciplines and encourage staff buy-in. In addition, available resources should be identified and made use of, especially staff e.g. those with educational or quality improvement roles. The TAKI study did not employ dedicated AKI nurses, but this practice is becoming more widespread and may offer another strategy to improve implementation. In addition, where identified enablers that may advantage some hospitals are missing (for example familiarity with package components), extra support could be provided, such as more time dedicated to demonstrating how the package is used and how easy it is to complete.

#### Time for embedding

Given the variety of potential barriers to identify and address, a longer period for embedding the intervention would be recommended. The complexity of the hospital setting means that barriers to uptake and fidelity may need further exploration during real-time implementation. In the case of TAKI, a three month implementation period was allowed; however qualitative data suggested that barriers were not restricted to this period despite the quantitative evaluation being unable to detect signal-changes throughout the period of implementation. Therefore, building in a method of regular identification or feedback on real-time barriers from hospital staff, throughout the implementation period and/or extending the embedding period, is advised. Fixsen et al (2005) [27] support this idea, suggesting that implementers, or ‘purveyors’ gain knowledge of barriers and solutions over time and recognise that this process can be lengthy (p14). A variety of consequences for staff, patients and their families has been shown to result from failing to identify and problem-solve such barriers [28].

## Conclusions

A variety of local factors–contextual, intervention-based and individual–were identified by stakeholders across the five hospitals as potential barriers and enablers for implementation of the TAKI package. They included both intervention structure/design and approach to implementation. Identifying, prioritising and addressing potential barriers is still a challenge, despite explicit advice in the literature and pre-planned attempts to do this. More extensive efforts to identify barriers prior to implementation are recommended, as well as real-time measures of implementation fidelity. In addition, optimising enablers such as strong leadership and multidisciplinary teams to encourage buy-in, and identifying existing expert staffing resources to collaborate in roll-out of the package would be advised. Combined, such efforts should reduce many of the identified, predictable and feasibly modifiable barriers to implementation, by similar teams in quality improvement contexts.

## Supporting information

S1 File TAKI implementing team interview schedule. (DOCX)

S2 File Framework analysis subthemes. (DOCX)
