# Supplementary material for: Barriers and enablers to the implementation of a complex quality improvement intervention for acute kidney injury: A qualitative evaluation of stakeholder perceptions of the Tackling AKI study
Source: PLoS One. 2019 Sep 20;14(9):e0222444. doi: 10.1371/journal.pone.0222444 (PMC6754144; doi:10.1371/journal.pone.0222444)
Supplement: S1 File — (DOCX) [file pone.0222444.s001.docx]

| 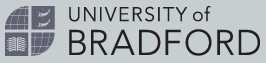 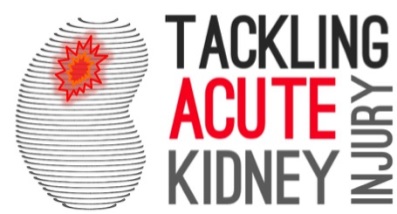 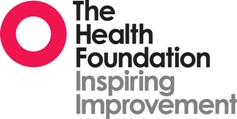  **Implementing Team Interview Questions for Tackling AKI project** | |
| --- | --- |
| **Participant: (tick one)**   \| **Frontline nurse** \| **Frontline doctor** \| **Project manager** \| **Clinical lead** \| **Lab/IT technician** \| **Pharmacist** \| **Education specialist** \| **Quality improvement specialist** \| **Expert patient** \| \| --- \| --- \| --- \| --- \| --- \| --- \| --- \| --- \| --- \| \|  \|  \|  \|  \|  \|  \|  \|  \|  \|   **Hospital:**  **Ward:**  **Study information:**  In your hospital there is currently a project running which aims to implement a package of interventions to help manage patients with Acute Kidney Injury (AKI). The package includes an AKI electronic alert, education of AKI and an AKI care bundle. The project aims to implement these in ward environments in five acute hospitals in four NHS Trusts, and aims to improve: patient outcomes and basic patient care.  As part of this project, we want to look at how this package of interventions has worked (or not), by finding out about any barriers or enablers to the design, implementation and delivery of them. We are holding interviews to investigate the views and experiences of key informants, who are familiar with the AKI intervention package.  Your personal details will be destroyed after collation of all the interviews. All information collected from you during the evaluation will be kept strictly confidential and stored securely. All information will be considered confidential other than in the unlikely event of disclosure of dangerous or harmful behaviour or intentions. Results will also be fed back to the implementation teams during and at the end of the project. No identifying information from any participant will be in any report or talk or publication. In fact, we will only report interview data across all the sites, rather than independently per sight, to protect participant anonymity.  [Give participant chance to re-read patient information sheet if they wish, ensure a copy of the consent form has been collected, if not, ask them to complete the consent form once again]  [If expert patient is being interviewed, show them images of AKI package as prompts as they may not have seen it recently.]   \| **Key Informant** \| \| \| \| \| \| \| \| \| --- \| --- \| --- \| --- \| --- \| --- \| --- \| --- \| \| **Frontline Nurse** \| **Frontline Doctor** \| **Project Manager** \| **Clinical Lead** \| **Lab/IT technician** \| **Pharmacist** \| **Education specialist/Quality Improvement Specialist** \| **Expert Patient** \| \| **1. Role** \| \| \| \| \| \| \| \| \| How would you describe your role in the AKI project? \| \| \| \| \| \| \| X \| \| **2. Overview** \| \| \| \| \| \| \| \| \| How do you think the AKI project (implementation and delivery) is going? \| \| \| \| \| \| \| X \| \| **3. Contextual Characteristics** \| \| \| \| \| \| \| \| \| What do you think it is about your hospital or ward that (has) helps/ed or hinders/ed the implementation of the AKI package of interventions?  [give broad examples of patient or staff involvement if necessary] \| \| \| \| \| \| \| As a potential patient, what do you think it is about your hospital or ward that could help or hinder the use of the AKI package of interventions?  [give broad examples of patient or staff involvement if necessary] \| \| To what extent have characteristics of your ward/hospital that could be changed, been addressed during implementation? \| \| \| \| \| \| \| To what extent have characteristics of your ward/hospital that could be changed, been addressed during implementation/use? \| \| **4. Design** \| \| \| \| \| \| \| \| \| Who were the key people involved in (a) the design, (b) implementation and (c) delivery of the package of AKI interventions and what did that involve? Were you one of them? \| \| Who are/were the key stakeholders involved in: (a) the design, (b) implementation and (c) delivery of the package of AKI interventions?  How have they been engaged in these three steps? \| Who were the key people involved in (a) the design, (b) implementation and (c) delivery of the package of AKI interventions and what did that involve? Were you one of them? \| \| \| \| Were you or other expert patients involved in (a) the design, (b) implementation and (c) delivery of the package of AKI interventions and what did that involve? \| \| **5. Intervention content** \| \| \| \| \| \| \| \| \| What is the package of AKI interventions? How has/do you know how the package of AKI interventions been: • developed • implemented • evolved over the course of the step wedge design \| \| \| \| \| \| \| Looking at the interventions, do you have any ideas about how these could have been introduced/used in the most effective way?  Are there any changes you would like to see in the package? \| \| **6. Theory of Change** \| \| \| \| \| \| \| \| \| How/Why do you think the AKI interventions and implementation works? E.g. how exactly is the package linked to the outcomes? What does the package do that results (or doesn’t) in better basic care and improved patient outcomes? • How has this method of working evolved over the course of the project? \| \| \| \| \| \| \| Do you think the package makes/will make any difference?  How do you think it works? \| \| **7. Acceptability and Utility** \| \| \| \| \| \| \| \| \| Do you find the AKI package of interventions acceptability and/or useful (three individual components and as a whole)?   - What are the perceived benefits, challenges, unintended consequences of the package? - What did you like the best about the package? - What did you like the least? \| \| \| \| \| \| \| Do you think the AKI package of interventions is acceptable and/or useful for patients (three individual components and as a whole)?   - What are the perceived benefits, challenges, unintended consequences of the package for patients? - What did you like the best about the package? - What did you like the least? \| \| **8. Barriers and Enablers** \| \| \| \| \| \| \| \| \| What do you think (has) helped or hindered implementation of the AKI package of interventions?   - What has been the biggest barrier? - What has been the biggest enabler? - How did you respond to these? - Does the design and delivery of the package of interventions address these? How and to what extent? \| \| \| \| \| \| \| As a potential patient, what do you think (has) helped or hindered implementation of the AKI package of interventions?   - What could be the biggest barrier? - What could be the biggest enabler? - How do you think patients might help or hinder implementation of the AKI package of interventions? - Does the design and delivery of the package of interventions address these? How and to what extent? \| \| **9. Shared Learning** \| \| \| \| \| \| \| \| \| X \| X \| What (if anything) have you learnt about scaling up quality improvement from the other hospitals? ( Might not apply to everyone, depends on how they have been involved) \| \| \| \| \| X \| \| **10. Sustainability** \| \| \| \| \| \| \| \| \| How is your ward/hospital approaching and planning for sustainability of the AKI package post-implementation? ( Might not apply to everyone, depends on how they have been involved) \| \| \| \| \| \| \| X \| \| **11. Buy in** \| \| \| \| \| \| \| \| \| How has your hospital approached ‘buy-in’ at different levels: senior exec, clinical leads, frontline teams? ( Might not apply to everyone, depends on how they have been involved) \| \| \| \| \| \| \| X \| \| **12. Engagement** \| \| \| \| \| \| \| \| \| How engaged do you think you are in the AKI package of interventions implementation and how do you think that compares to other people? \| \| \| \| \| \| \| How engaged do you think you (or other potential patients) are in the AKI package of interventions implementation and how do you think that compares to other people? \| \| **13. Removal of AKI package** \| \| \| \| \| \| \| \| \| If the package of interventions was taken away tomorrow, would you (or a patient) miss it and why? \| \| \| \| \| \| \| \| \| **Total number of questions asked** \| \| \| \| \| \| \| \| \| 12 \| 12 \| 13 \| 13 \| 13 \| 13 \| 13 \| 8 \|   **14. Is there anything else you would like to add?**  [Re-clarify anonymity and data reporting as said above]  [Offer participants copy of results when available.]  [Thank participant for their time]  **END OF INTERVIEW** |  |
