# Supplementary material for: Barriers and enablers to the implementation of a complex quality improvement intervention for acute kidney injury: A qualitative evaluation of stakeholder perceptions of the Tackling AKI study
Source: PLoS One. 2019 Sep 20;14(9):e0222444. doi: 10.1371/journal.pone.0222444 (PMC6754144; doi:10.1371/journal.pone.0222444)
Supplement: S2 File — (DOCX) [file pone.0222444.s002.docx]

**S3: Framework analysis subthemes: Exemplar quotes demonstrating full set of themes related to barriers and enablers for implementation of the package of interventions**

Numbered themes relate to those key themes in main report table. Additional themes not represented in the main report are unnumbered.

| **Theme 1: Practical and contextual factors** | |
| --- | --- |
| **Subtheme - Enablers** | **Exemplar quote** |
| 1. Key staff/team availability and capacity | “…the things that have really made a difference between the different centres was whether or not there were established teams to actually go out and target ward staff either from an educator’s point of view or from a sort of quality and patient safety point of view. The hospitals that had those teams already established and in place were very much more set up and able to do that than those that weren’t.” PN:28 |
| 1. Ward or hospital structures, context or goals/attitudes | “…patient safety is at the heart of what I do, and I would say that it’s really important to the organisation“ PN:17 |
| 1. Senior staff, or staff support and involvement in general | “..you know, [Senior Clinical Executives] philosophy is one of openness and you can approach them. Anybody can drop them an email about stuff and, you know, if it’s not them directly, they will get somebody to speak to you so that they know that is what’s happening. They’re great. You know, if I say, “Open door?” Definitely have an open door. Very supportive.” PN:14 |
| 1. IT systems | “ …and we had the ICE system the laboratory system which is very configurable so we didn’t have to wait for some national or international group to make changes to our results server we were able to do a bespoke modification of it so we could link the e-result, the e-report, the e-alert I should say with that guidance by just clicking on the resources tab within the results server and [person’s name] was able to whatever we agreed as content we could just literally say yeah that’s the content we want and then we could upload it as finals within that resources tab and it was sort of a living document that we could modify in [hospital] for [hospital] without having to go begging for a national body to make changes to it,” PN:2 |
| 1. TAKI project related structures and material resources | “…local care bundle finalised, being sent to printers imminently. Funding in budget to cover printing costs. Will be printed on a sticky label that can be stuck into hospital case notes” PN:37a |
| 1. Hospital familiarity and acceptability of similar intervention tools or approaches | “…we’ve already got a sepsis care bundle which is very similar to this one which is what we wanted because that’s been very successful the whole hospital is aware that there is a sepsis bundle so if they’ve got a patient they think is sepsis they tick-box them and plonk this thing in the notes therefore having that beforehand meant that when we came along with the AKI one with the similar format that was very useful because people knew, you could say actually you do it like the sepsis one and then we linked it” PN:22 |
| **Subtheme - Barriers** | **Exemplar quote** |
| 1. Limited or lacked awareness of material resources or services | “…as we’ve all found the resources we’ve tried to implement have been quite tight and we realise that a lot of the Trust[s] have used some of the funding that they’ve had for AKI nurse specialists but they then had a big Trust… with lots of people and lots of money and you’ve got lots of people that can you know come and help to support the project….[Project Team] really struggled to get the education out and to keep on top of the audits with the resource that we had.” PN:31c |
| 1. Systemic changes (e.g. IT systems, ward changes) | “…they merged on the [DATE] and then the nurses that we’d spent a lot of time with on [ward 1] they all went to [ward 2]! All of the nursing staff on [ward 3] came over onto the new ward… So we basically had to start again before the new staff were on there.” PN:31c |
| 1. Characteristics of the Trust, hospital, ward or staffing structures | “Matrons seem to have a different role there to the Matrons I’m used to working with at other organisations in terms of they don’t appear to be so hands-on, on the ward and they don’t seem to have any interest in what’s happening in their clinical areas, I’m sure it’s just because they have a different role” PN:1 |
| 1. IT systems and processes | “…but in that initial guidance the AKI alert was only for inpatients not for primary care, not for GP patients, now ideally what we wanted to do was carry on producing the alert for primary care for gathering some data but because of the limitation of the system we couldn’t generate the alert for the inpatients and stop for the GPs at the same time, its either done for all patients or so we had to sort of come up with a fix which was that for all GPs we weren’t reporting the actual staging, we were just saying ‘NA’, and NA sort of covered other scenarios as well so for example if there's a problem with the sample or if the [Inaudible 00:11:41] was excluded so for example in neonates or dialysis patients it gives the same staging NA, so we just had to come up with a comment which would cover all the scenarios, so ideally the way we would have designed basically if the system allowed us to have a separate comment for each of so for example for dialysis patient we would have said AKI not calculated because this patients on dialysis or for a neonate an AKI is not calculated because of the age, and then likewise for the GP patients” PN:4 |
| 1. Busy care marketplace or current Trust priorities | “I think it’s a busy marketplace – there's lots of [investment] around dementia screening, around patients who need to have special protection adults and so forth, the alcohol dependency questionnaire; there's so many things you’ve got to get right in admissions that it’s a busy marketplace to get the attention on the admitting team” PN:2 |
| 1. Repetitive approaches to Quality Improvement | “Well we did look at champions didn’t we but then they’re a bit championed out really because there's [Inaudible 00:36:42] champion, resus champion erm dementia champion and you get to another one and they look at you as if to say ‘really, another one?’ so it’s quite difficult…” PN:31a |
| 1. TAKI project arrangements or structures | “And the distances (inaudible 0:22:33.5) that was just opportunities really because obviously with [Hospital sites], we’d have loved to spend more time actually going to see but that's just because of the way…, we're a hell of a long way from each other, aren’t we in terms of getting to see each other” PN:35c |
| **Theme 2: TAKI Project Team** | |
| **Subtheme: Enablers** | **Exemplar quote** |
| 1. Multidisciplinary team, mixed skill set including specialist skills relevant to TAKI project | “…so I think it’s been a good sort of melting pot for ideas and sharing of thoughts around how things can be done differently with good interactions with the whole of the [Multi-Disciplinary Team]” PN:29b  “I think having the support from the Improvement Academy I think that was a great help because obviously they’re the sort of project manager type, the side that I’m not really used to.” PN:16 |
| 1. Involved in groundwork prior to TAKI | “…the core medical trainees so I thought that was a good group of trainees to give us a lead on what sort of content they’d like to see when they got an e-alert popping up so it was from that sort of workshop type activity that had preceded the project that we had some idea of what we wanted to go into the care bundle” PN:2 |
| 1. Had established links or networks internal or external, relevant to TAKI | “ [Team member] is involved in kind of like national projects on AKI” PN:17 |
| 1. Team enthusiastic, proactive/autonomous, visible | “…the enthusiasm of the clinical leads we’ve got is a rare event. To have somebody with that passion/drive/internal connections and credibility is even rarer, so a clinician who can influence other clinicians is like hens’ teeth, they usually can influence with their own field, but influencing outside their own field is really difficult so it takes someone with a very specific type of communication skills to be able to do that and fortunately we’ve got people in the team that can do that, who are good influencers, and that’s been hugely necessary and valued really within the work…..And then again we’ve just by chance picked up people like [name] who is passionate and is able to link with the [specialism] teams” PN:1 |
| 1. Regular forums for team | “I think the monthly meetings are really good, it kind of blocks off a piece of time for supervisors and so on… you can at least get your point across just for that hour in a month which is good “ PN:24 |
| 1. Team cohesion and support | “Likewise, [Name] and I developed a really positive working relationship together. So, even though we were both busy, we really felt like we were supporting each other and working together.” PN:14 |
| 1. Team members have dedicated time | “I think we had enough people to go round for the…[pilot ward/s] we had at that time….We had enough resource…” PN:16 |
| 1. Strong/good team leadership | “So the clinical leadership has been fantastic” PN:1 |
| **Subtheme: Barriers** | **Exemplar quote** |
| 1. Team members absent or change (includes who was sought for membership as well as how member engaged) | “…it’s just that nurse bit again, the nurse educator bit which is what we’ve lost, it’s a bit devastating really and we’ve never managed to replace it.” PN:1 |
| 1. Team size and capacity (QI is resource intensive/time consuming) | “Collecting stories is motivational, and well received, but it is time consuming. The stories are powerful and worthwhile. There are lots of ways in, but [Communication Team] need persuading to publish them.” PN:35a  “So the challenges then were really its very time consuming to do quality improvement work, none of us really had dedicated time for this” PN:29b |
| 1. Team dissent | “…it was difficult to get total agreement from everyone involved as what was actually in the [care bundle], so that has meant that we have had a number of guidelines written…” PN:26 |
| 1. Poor team management | “…we didn’t really have much direction from our lead” PN:10 |
| 1. Lack of team authority, knowledge or skills | “…here I have to rely on, you know, [other team members]... which is fine but it just means it's another step so for me I was thinking this isn’t as easy when you don't actually have that sort of ... not even positional power route, just influence and people don't know me.” PN:35b |
| 1. Hospitals staff perceptions of team | “When an [specialist team] get involved with these sorts of projects, I think the barriers are always going to be that we are highly selective as to which patients get in and out of [ward] because of the resource capacity and the resource issues we have, and so a barrier is already set up in that there’s a belief from the physicians that, “You don’t take our patients, so if you’re running a project why should we necessarily listen to what you have to say?” And I think that is a very real barrier, that I don’t think if you have on-site renal physicians and renal physicians leading this AKI project, if it’s coming from a cohort that you are highly involved with who attend a lot of your divisional type meetings, I think it’s more powerful.” PN:11 |
| 1. Lack of team cohesion (feelings of unit, feeling part of a group) | “Sitting on the committee I sometimes feel as if, “What the hell am I doing here?” I feel I haven’t been involved very much and I sometimes think, “Well I’ve taken an hour of my day or an hour after work – why am I bothering?” As I say, there’s been a couple of things that people have said, “Oh yeah, we’ll contact [name] and ask [name] about,” and I haven’t heard anything.” PN:19 |
| 1. ‘Hearts and minds’ not convinced of package or implementation process | “Well first of all I’d like to say, you know, it’s a very worthwhile thing. I hope it shows that it’s useful to have a bundle. You know, I’ve read some of the literature, published literature on it and I must say I’m a little bit sceptical, so will it work or not. But I hope it does and I hope that we can continue the work in our Trust and I hope that we get the sustainability.” PN:6  “I think we had a [team member] who perhaps didn’t feel the love for it, it would be fair to say, but I think that’s because they ended up having to do [certain project tasks] and I think that didn’t necessarily motivate [team member]. So [team member] dropped out of the project, which was a real shame.” PN:14 |
| **Theme 3: Design, development and implementation approach** | |
| **Subtheme: Enablers** | **Exemplar quote** |
| 1. Approach aimed to facilitate frontline staff engagement and ownership of the package (involve staff in design, championing delivery, success celebrations, public communications,) | “…we’ve used champions, we’ve used the champion model, we’ve gone round and tried to find people; we have two new champions to help with the spread of the new wards… she was involved in the project right at the beginning and…came back to Bradford to be a registrar on an [pilot ward], …so she was perfect, in place and knew everything about the project already and wanted to help. The other guy was a slightly different route, because [Name] …had been out and about had talked to some of her colleagues and said, “Would anybody like to help on the surgical ward?” And we’d come up with this guy…” PN:1 |
| 1. Approach engaged with and tailored to hospital structures, ethos, characteristics | “Well I suppose the best thing was like the things where they’ve taken a bit of shaping I suppose cause they are therefore shaped to fit these things on purpose, they’re not off the shelf so I suppose the best bit about it has been all those elements of the packages have needed some form of tailoring, to fit the needs of the local trust, making it as appropriate as possible.. “ PN:2 |
| 1. Approach promoted team-based delivery and learning | “…and shared the project with them and to try and get some engagement which was an interesting useful meeting, so it’s really about saying we’re here, this is what we’ve learnt, we want to come and work with you, again those key messages around working and learning together…” PN:29b |
| 1. Pre/during-implementation preparation | “…so those who have governance responsibilities, leadership responsibilities in those areas have made a commitment from the start of the project that they would be ready and waiting for receiving a sort of summary of progress on the [pilot ward] and run with it in this second phase so we’d kept the pot boiling with discussions in that respect…” PN:29b |
| 1. Implementation approach included feedback to promote project legitimacy and staff engagement | “That was supporting with the real-time data collection, the purpose which was more round engagement than actually collecting accurate data, but the two things kind of went hand in hand in that we found something wasn’t working it gave us a structure for a conversation to try and understand a little bit why, and help us answer the next question really, and then led to quite substantial changes being made to the care bundle in particular and also some changes from the education packages and things in terms of some of the feedback we’ve had from the ward team.” PN:29b |
| 1. Quality Improvement approach to implementation | “It’s kind of backed up what I understand about spread and what I’ve learnt over the years working improvement around spread is that you do start small and you do concentrate your resources and you do make yourself a nuisance and you are there every day and you ask the idiot questions and you get what you need out of it….” PN:1 |
| 1. Tailored approach and content to frontline staffing groups | “…and then the clinician to clinician works well…” PN:1 |
| 1. Shared learning from other centres | “…so I suppose that’s been the benefit in terms of sharing what we’ve developed in one trust with others so we’re not having to reinvent the wheel, so for example we shared our care bundle with the other trusts and we share some of their information,…” PN:4 |
| 1. Persistent implementation approach | “…so we’re starting again going back to them and saying, “Look, how are you getting on?” and “Do you know how you find out how many you’ve got?” or maybe go to them and say, “You know, did you know you had ten last week?”…” PN:8 |
| 1. Engagement with senior executives | “…there is a good relationship between the Quality Team and the Medical Director and he’s been very engaged as well, so there is a free-flow of information backwards and forwards, but he’s hearing barriers from all different types of improvement projects, not just ours, so it’s how to help them process that and work out what fits with other things to get things done.” PN:1 |
| 1. TAKI linked to other or previous projects/work /initiatives | “One of my worries is that people will get bored about it; I don’t think they are, I think they embrace it, they understand that it’s not a kidney thing, it’s not a nephrologist banging on about a kidney thing it’s more than that, it’s to do with a vulnerability in a patient, it’s to do with a patient who happens to develop acute kidney injury because something else has happened, whether it be sepsis, that fits in with sepsis [CQUINs], and the requirements the Trust have to meet that…. It fits in with prescribing the pharmacist so it cuts across a number of different areas, and then it’s also around medicines management and fluid management…” PN:12 |
| 1. Health Foundation affiliation | “Health Foundation funding, badging with Health Foundation funding, very, very important because it gives it kudos and credibility, when you say half a million, ker-ching, ker-ching, is behind this project it’s also got credibility there.” PN:12 |
| 1. Extra information and support materials beyond core package items | “…we’ve got pens/post-its, all that stuff has now arrived, and that helps when you’re going on a ward, because if you just take out pads and pens everybody wants to talk to you, it’s ridiculous but it’s true.” PN:1 |
| 1. Alert characteristics | “…it helps everybody to sort of I suppose fulfil their responsibilities as well in terms of so from the labs point of view flagging results up although we were picking out raised creatinine’s but if say the result had changed from 90 to 160 we may not immediately of the significance but if it’s an AKI stage two then it’s just a lot easier to know its implications.” PN:4 |
| 1. Care bundle characteristics | “Probably the fact it’s easy to use, the fact it’s just one big sticker and it was very few questions but had the right information that needed to be delivered“ PN:3 |
| 1. Multi-disciplinary nature of package | “…[Nurses are] not used to looking fully at the bloods and seeing what level they’re at and what treatment whereas with the AKI it tells everybody that information rather than the medical team getting so much information and the nursing team getting so much and working differently it now brings the two together and they work together so a nurse then therefore can say well actually I’ve just spotted that this patients an AKI and they need some fluid and they’re actually prescribed this can you cross it off, so the actual two work together rather than waiting for a doctor to spot it, a nurse to give the medication cause they didn’t know the patients got an AKI and then the doctor later on to say well this mans had this, I think it actually brings the two together.” PN:3 |
| 1. Education characteristics | “And it also comes back to an educational thing isn’t it I mean fluid balance isn’t always easy than sometimes when things aren’t black and white it’s harder to commit to them and write them down in the notes so that’s part of the education which goes a little bit beyond AKI and links through to other things as well.” PN:29b |
| 1. Multiple methods of engagement with package | “[Junior doctors] are a challenging group and it’s difficult to get them all together, so [team member 1] done teaching with the F1s and F2s and [team member 2] done teaching with them as well, our renal register.” PN:10 |
| 1. Perceived efficacy and utility of package | “I certainly found it very...I think it is a very worthwhile project because of the severity, the serious nature of AKI.” PN:7 |
| **Subtheme: Barriers** | **Exemplar quote** |
| 1. Spread plans incompatible with project team resources | “…and then when you go to the next ward in theory it’s easier, but then it gets more difficult because you’ve got less people so they’re not going to get the same level of support that you’ve got, and that’s when things like the education and the resource for education comes into play and if you haven’t got it that really slows you up.” PN:1 |
| 1. Limited access to and involvement of key staff | “…they have regular teaching sessions, the F1s and F2s, but the registrars don’t you see, so it’s difficult to get this information disseminated to the more senior doctors; the registrars don’t, it’s difficult to get to the consultants as well, the consultants, apart from (inaudible 00:10:48) either, you don’t have to, it’s a voluntary thing; they just turn up for the free food and then they go away again.” PN:10  “I: Were [frontline teams] involved during design and implementation do you know?  P: Not really no, not so far as I’m aware, that was more … well, there was some feedback once the pilot wards were involved, obviously there was some feedback then and there were some tweaks made, I can’t remember the specifics but there were some.” PN:13 |
| 1. Package characteristics (visibility, availability, accessibility) | “…so you’ve got to go looking for it, it isn’t really an alert, it’s a detection, a facilitator really so you’ve got to go looking for the results to know that its abnormal,…” PN:2  “I think [the education is] really good to start with, I probably would have liked maybe more throughout.” PN:3 |
| 1. Package adds to workload | “We do have a lot of other sort of pieces of papers and projects and flow charts and all that sort of thing (inaudible 5:53) and all that kind of thing. So I think other people felt it was another form that they had to fill out and another bit of paper they had to put in the notes, so it was sort of trying to make it standout from all those other bits of paper as well.” PN:9 |
| 1. Package characteristics (inappropriate/ insufficient content) | “…so the heart failure people, the people that don't have enough pump you can't give them too much fluid but the AKI bundles for everywhere…so you just give them fluid so it’s a very small population group,…, but then if you just blindly follow the AKI bundle which is kind of what it’s there for, it’s there to save the people that you know if you don’t follow it they’re going to die more and that’s true but if you blindly follow it you’ll get your heart failure that you…a bit that would be a small amount of fluid in them in a normal person but because their hearts are failing it’s a lot of fluid and then they get overloaded with fluid because they’re not able to push it round their body like they should and then they have to come to the intensive cardiology unit to help offload that fluid, so it’s just kind of those tweaks, it’s the smaller percent of the population that don’t fit the algorithm and its knowing how to highlight that to people cause it’s a great algorithm per say but not everyone will fit it.” PN:24 |
| 1. Package neglects some staff involvement/ is inappropriately exclusionary | “The one thing I find a little frustrating is that it’s very medically focussed and I think that we have a lot of advanced nurse practitioners and things that I think could sign-off some of this stuff.” PN:14 |
| **Theme 4: Hospital staff knowledge, attitudes, behaviours and support** | |
| **Subtheme: Enablers** | **Exemplar quote** |
| 1. Staff support and engagement | “Other groups of staff like the pharmacy team have been fantastic: really engaged and really wanting to work out actively what their role is going to be and how they can support the work.” PN:1 |
| 1. Champions or proactive and receptive wards and teams | “…the advantage there around staff being able to take already messages back to parent wards that includes FY1’s who are doing medicine at the moment who are about to move onto surgical rotations so that quite a few have been speaking to me already saying that they’re going to or wanting to be part of the rollout in these other areas that they’re moving to subsequently.” PN:31c |
| 1. Nurse attitude and presence | “Probably our attitudes and the drive of the other nurses. Just because they’ve not given up and I’ve badgered them week in week out making sure we do it. I’m like, “guys if there’s an AKI we get that sticker out, we get the file out, you get your little cards out, you do what you need to do.” Yeah, I’d say probably our nursing attitude towards it just because I know at the minute how stressed and stretched out we are, but still, you know, they get on with it and everyone has just got on with it and done as much as they can in their hands and power that they could do at that time to be fair.” PN:18  “I think it’s really good that we’re getting into the nursing profession, they’re really embracing it because they will be the framework I think around … the doctors will change again won’t they in August, so I think the more we can embed it there, will start to seep through into others. We would like to make a bigger impact right now wouldn’t we, but I think we’re feeling these sorts of things take a while to change a whole culture, but if the nurses are taking it on as a cultural change I’m really encouraged by that longer term.“ PN:34b |
| 1. Multi-disciplinary package and multi-staff facilitation of package use | “I see nurses prompting doctors to fill out the AKI forms, I’ve seen pharmacy comments on front of drug charts recommending changes in drug doses or stopping drugs, I’ve seen lots of involvement.” PN:26 |
| 1. Senior and executive support and engagement | “…through the grand-round I invited [Medical Director] to come to the grand-round as well to be supportive, and also he helped with the AKI CQUIN letter so I drafted a letter for him to send out to remind all staff to remind their juniors to complete the AKI CQUIN discharge summary so [Medical Director] been very helpful at all levels of the project in that respect, he’s also promoting the mandatory training element.” PN:2 |
| 1. Understanding and acceptability of package and role in completing it | “I think that people are starting to recognise … to start with it would have, but I think they’re actually recognising what the acute kidney injury means, as we’ve got more information across generally through ‘Think Kidneys’ through the fact that kidneys are important organs, that we do need to engage with them, that there are risks associated with kidney disease, acute kidney injury of death and chronic kidneys, it’s not just this thing where a number goes up and a number comes down…I think when people start to realise they can do something about it, rather than referring to a nephrologist and waiting for them to come 2 days later because they’re so busy seeing everyone else, that is a strong motivator.” PN:12 |
| **Subtheme: Barriers** | **Exemplar quote** |
| 1. Hospital staff fail to engage | “…some of the consultants that we’ve been inviting to come from the beginning haven’t realty turned up to be fair so we’re trying to engage them and get them to come, the renal registrar has but again its quite difficult with the timings and their workloads to get everybody to come.” PN:31c |
| 1. Failure to see utility of package/biased perception of behaviour | “I think the problem comes where...because obviously a lot of us, we are newer doctors, and we are much more open to change, and we don’t have expectations that have been there for years, whereas in a lot of the older doctors, not necessarily consultants, but registrars and more senior SHOs who are people from their second year up until their fifth year, in those people they have been practicing medicine for quite a lot longer, and maybe don’t feel as though the bundle is necessary.” PN:7 |
| 1. Perception of frontline staff responsibilities around AKI | “I have presented to [senior nurses], they know about the project but I don’t think they see their role as particularly delivering this.” PN:1 |
| 1. Failure to use infrastructure as expected | “Also how well the ward use the whiteboard, whether they look at the prompts, we also had to make some quite big arrows that we stuck on the whiteboards that said, “Look at AKI,” to almost prompt them to look at the board to look at the column. I think originally they thought that the doctors would look at the whiteboards maybe a lot more than they do, so that was one of the barriers that we found was that they wouldn’t always look at the column to see that the patient had an AKI from looking at the whiteboard,…” PN:16 |
| 1. Senior staff ‘role models’ | “So, I think, depending on which consultant you’re working with, that affects how much you are using the AKI, kind of, toolkit. For example, one of the consultants who I work with on [ward], if there is an AKI alert; she will stop and go through it, whereas on the acute floor, particularly when it’s very busy, the consultants won’t do that. As a junior, you’re also very busy doing lots and lots of jobs. You normally don’t, kind of, get the time to get around to doing it. So, I think it depends a lot on where you’re working and the seniors that you’re working under.” PN:20 |

***Definitions:*** *I: Interviewer, P: Participant, PN: unique data source number, []: Data has been changed for confidentiality purposes (e.g. names removed), or clarified for ease of understanding (e.g. ‘it’ replaced with AKI package element being referred to)*
